# Assessment of the Refractive Index and Extinction Coefficient of Graphene-Poly(3-hexylthiophene) Nanocomposites †

**DOI:** 10.3390/polym14091828

**Published:** 2022-04-29

**Authors:** Lara Velasco Davoise, Rafael Peña Capilla, Ana M. Díez-Pascual

**Affiliations:** 1 Universidad de Alcalá, Facultad de Ciencias, Departamento de Química Analítica, Química Física e Ingeniería Química, Ctra. Madrid-Barcelona, Km. 33.6, 28805 Alcalá de Henares, Madrid, Spain; lara.velasco@uah.es; 2 Universidad de Alcalá, Departamento de Teoría de la Señal y Comunicaciones, Ctra. Madrid-Barcelona, Km. 33.6, 28805 Alcalá de Henares, Madrid, Spain; rafael.pena@uah.es

**Keywords:** nanocomposite, refractive index, extinction coefficient, graphene

## Abstract

Poly(3-hexylthiophene) (P3HT) is one of the most attractive polymeric donor materials used in organic solar cells because of its high electrical conductivity and solubility in various solvents. However, its carrier mobility is low when compared to that of inorganic semiconductors; hence, the incorporation of appropriate nanomaterials to improve its electrical mobility and optical properties are pursued. In this work, a review of the changes in electrical conductivity, bandgap, hole collection properties and carrier mobility of P3HT when adding graphene (G) is presented. The main aim is to assess how the addition of different G contents influences the optical constants: refractive index (*n*) and extinction coefficient (*k*). The values of *n* and *k* as a function of the wavelength for six P3HT/G nanocomposites with G loadings in the range of 0.1–5 wt% have been fitted to two different models, Forouhi Bloomer and Cauchy, showing very good agreement between the experimental and the theoretical values. Furthermore, a rule of mixtures was successfully applied to calculate n using mass fraction instead of volume fraction, with errors lower than 6% for all the nanocomposites studied.

## 1. Introduction

Over the last decades, electricity production via renewable resources has grown extraordinarily due to the increasing concern about the impact of fossil fuel-based energy on global warming and climate change [1]. In this regard, photovoltaic energy can contribute to environmentally friendly and sustainable energy production. The different photovoltaic cells developed up to date can be classified into different technologies or generations [2]. Amongst them, one of the most currently investigated is organic solar cell (OSC) technology, which uses conductive organic polymers or small organic molecules for light absorption and charge transport to produce electricity from sunlight by the photovoltaic effect. OSCs have attracted considerable attention in the past few years since they are environmentally safe, flexible, lightweight, and inexpensive [3]. They typically comprise an electron- or hole-blocking layer onto an indium tin oxide (ITO) conductive glass followed by an active layer with an electron donor and an electron acceptor, a hole or electron blocking layer, and metal electrode on top. The nature and order of the layers strongly condition the device performance and, consequently, their efficiency. These cells can follow a regular or an inverted device architecture [4]. Inverted OSCs typically display longer lifetimes and higher efficiencies than their conventional counterparts. In this context, the efforts are mostly concentrated on increasing power conversion efficiencies and reducing the cost of materials and processing conditions.

**Figure 1:**
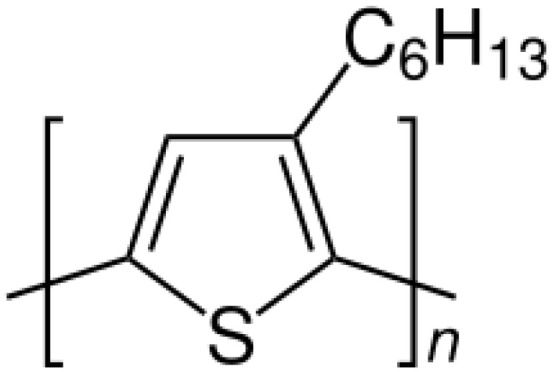
Scheme of the P3HT monomeric unit.

A wide range of polymers has been used for the manufacture of OSCs [5,6]. Poly(3-hexylthiophene) (P3HT) is a regioregular polymer, which means that it contains only head-to-tail (HT) couplings between monomers. The monomeric unit of P3HT is shown in Figure 1. It is a p-type semiconductor and is one of the most attractive polymers as donor material due to its outstanding chemical and electrochemical stability, high electrical conductivity and an appropriate HOMO energy level of approximately −4.9 eV [7]. Moreover, P3HT has a high absorption coefficient (on the order of 10^5^ cm^−1^) and can absorb more than 95% of the solar spectrum over a wavelength range of 450–600 nm when deposited as a thin film. This makes it very attractive for organic optoelectronic devices [8]. It also presents several other advantages such as solution processability, easy and low-cost fabrication, and exhibits one of the highest electrical mobilities amongst those of known conjugated polymers, of about 0.2 cm^2^/(V·s). However, this mobility is low when compared to those of inorganic semiconductors [9], which results in a low photocurrent. In this regard, the inclusion of appropriate filler nanomaterials such as metal oxide nanoparticles [10] or carbon nanotubes [11] has resulted in significant improvement in its electrical mobility and optical properties.

On the other hand, new acceptor carbon-based materials such as graphene (G), a one-atom-thick sheet of sp^2^ hybridized carbon atoms that was first discovered in 2004 by Novoselov et al. [12], have been widely analyzed due to their exceptional electrical, optical, chemical, and mechanical properties. Graphene has an extraordinary electrical mobility of 15,000 cm^2^ /(V·s), a high specific surface area (ca. 2600 m^2^/g), very high electrical conductivity (up to 6000 S/cm) and transparency higher than 70% over the spectral range of 1000–3000 nm [13]. A single sheet of graphene has extraordinary mechanical properties because of its honeycomb-like hexagonal structure in which the carbon atoms form a perfect crystal lattice. Thus, it is one of the strongest materials on earth, with a tensile strength of 130 GPa and Young’s modulus close to 1 TPa [14]. However, the very low responsivity due to its weak light absorption and fast recombination rate has limited the sensitivity of graphene light-sensing devices [9]. Significant efforts have been applied to increase its absorption, among which a feasible way is the combination with light-absorbing materials [15].

Due to the unique optoelectronic properties of P3HT and graphene, the combination of these materials offers the opportunity to obtain an improved nanocomposite in terms of performance. Particularly, a high absorption coefficient (like that of P3HT) and a high mobility (like that of graphene) are possible in P3HT/G nanocomposites.

Several methods for the preparation of P3HT/G nanocomposites have been reported, including solution mixing and in situ polymerization [16,17]. The in situ polymerization consists in mixing nanofillers with a liquid monomer or a precursor of low molecular weight. When a homogenous mixture is attained, polymerization is initiated by the addition of an appropriate initiator, which is exposed to a source of heat, radiation, etc. [18]. Polymerization is then carried out by adjusting the temperature and time. It is a very effective method that leads to a uniform dispersion of carbon-based nanofillers within the matrix, thereby providing a strong interaction between them [17]. However, certain conditions must be fulfilled, including the use of low viscosity monomers, a short period of polymerization, and no formation of side products during the process.

Changes in electrical conductivity, bandgap, hole collection properties and carrier mobility of P3HT when adding G have been previously studied by a few authors [8,9,19,20,21]. In a pioneer work, Chang et al. [8] explored the optoelectronic properties of P3HT/G nanocomposites prepared via in situ reduction of exfoliated graphite oxide in the polymer matrix. An improvement in electron mobility and electrical conductivity was found with increasing G loading. Their conductivity measurements for different G concentrations are shown in Table 1. It is noteworthy that a significant increase can be observed, from values of 9.273·10^−3^ S/m for pristine P3HT to 1.878·10^−2^ S/m for a G loading of 10 wt%.

Saini et al. [9] investigated the structural and optoelectronic properties of P3HT/G nanocomposites developed by in situ oxidative polymerization of a 3 hexylthiophene monomer in the presence of graphene. Yadav et al. [19] fabricated an organic photodetector with solution-processed P3HT/G composites. The incorporation of G caused a red shift in the UV-vis absorption spectra of the polymer, indicative of an increase in the conjugation length. The photocurrent was enhanced in the presence of this nanomaterial, and the device with 5 wt% G showed the best electrical performance. The same authors [20] recently explored the effect of annealing temperature on the charge injection and transport properties of devices with these nanocomposites as the active layer. An increase in annealing temperature resulted in a significant improvement in electron mobility. Shariff et al. [21] developed the same nanocomposites via spin coating, and the optical properties such as absorbance, transmittance and photoluminescence were significantly improved compared to pristine P3HT, showing the optimum performance at a G concentration of 2 wt%.

The bandgap of P3HT is around 1.9 eV, which limits the absorption of wavelengths under 650 nm. In this spectral range, and under AM 1.5G standard spectrum [22], only 22.4% of the photons are found. Consequently, in OSCs with P3HT as the active layer, decreasing the bandgap leads to an increase in the total amount of photons that can be harvested. However, narrowing the polymeric bandgap results in a reduction in the power conversion efficiency of the cell due to a decrease in the open circuit voltage (*V_oc_*). Therefore, a trade-off should be achieved to obtain the optimum bandgap [23].

According to Bkakri et al. [24] and Chang et al. [8], spectroscopic ellipsometry (SE) analysis proves that the insertion of a low G content in the P3HT matrix reduces the thickness of the film and the optical bandgap of the P3HT/G nanocomposites. As a result, the optical absorption properties of the solar cell with these nanocomposites in the active layer improve in the visible range. Nonetheless, according to Saini et al. [9], who used optical spectrophotometry (UV-Vis-NIR) measurements, there is a lack of a clear trend in the change of the P3HT bandgap with the G content. There seems to be a slight increase in the bandgap for low G contents, showing a maximum at 1 wt% G. However, for higher G loadings (up to 10 wt%), both the absolute values of the HOMO and LUMO levels slightly increase so that the bandgap keeps merely unchanged. Therefore, it is necessary to clear out this discrepancy between authors about the bandgap, which likely arises from differences in the measurement techniques and in the processing conditions. Indeed, the device performance relies strongly on the surface morphology and the presence of structural defects, which are inherently formed during the film processing, as well as on the nanofiller-matrix interfacial properties, which eventually determine the charge transport and collection.

On the other hand, the use of P3HT/G improves the efficiency of hybrid solar cells due to the enhanced hole collection of the polymer in the presence of graphene [25] (the incorporation of a 5 wt% P3HT/G nanocomposite increased the efficiency of solar cells with ZnO nanowires from 0.09 to 0.4%). P3HT/G bulk heterojunction prepared by solution processing combines the advantages of the high carrier mobility of G and the high visible light absorption of P3HT. In this regard, Che et al. [26] fabricated a phototransistor consisting of a solution-processed P3HT/G bulk heterojunction channel. The device exhibited a hole mobility as high as 3.8 cm^2^/(V·s) due to the enhanced charge transport properties of G.

The abovementioned previous studies have measured the optoelectronic properties of P3HT/G nanocomposites using different experimental techniques, although some contradictory results have been attained. Therefore, with a view of using these nanocomposites in photovoltaic applications, it is essential to clarify these discrepancies. In this regard, the present work aims to assess how the main P3HT optical properties behave or change in the presence of different G contents using theoretical models that are not influenced by the experimental method. In particular, the refractive index (*n*) and the extinction coefficient (*k*) of different P3HT/G composites are shown, and the influence of G content on these parameters is discussed. n and k of the nanocomposites with G concentrations from 0 (P3HT) to 5 wt% have been fitted to the Forouhi Bloomer and Cauchy models with the aim of obtaining theoretical expressions that describe n and k and simultaneously have physical meaning. There is a lack of theoretical studies on how the graphene content influences the optical properties of graphene/polymer composites. To the best of our knowledge, no previous study on the modelling of the optical properties of P3HT/G nanocomposites has been previously reported.

## 2. Methodology

The complex refractive index of P3HT has been studied before and after the addition of G. For such purpose, firstly, an exhaustive bibliographic search has been carried out. Special attention has been paid to those results related to the application of the studied compounds to organic solar cells.

Then, a software tool to fit n and k to different analytical models was developed. Forouhi Bloomer, Cauchy and other models have been evaluated, and the first two were finally chosen because they lead to lower errors in the fits [27].

The curves in Reference [9] have been used as the source for the experimental data. In this regard, the values for n and k have been selected from those curves registering equally spaced points in a database processed using Matlab R2020b (MathWorks, Inc., Natick, MA, USA). The spectral range considered is between 4100 and 11,100 Å with an interval of 200 Å between registered points.

The registered data have been fitted to the aforementioned analytical functions. The values chosen for the fit parameters are those for which the root mean square error (RMSE) for the calculated n (or k) is minimum compared to the experimental data. An exhaustive search optimization approach is used for this purpose, as follows:

(a) A search interval is estimated for each parameter. Each interval is supposed to contain a guess for each parameter.
(b) The exhaustive search is applied for the intervals selected in (a). In this step, a first solution for the fitted parameters is obtained. As shown above, the selected parameters are those for which a minimum in the RMSE is obtained.
(c) The search intervals for each parameter are reduced. Each new interval must be centered in the parameters obtained in (b).
(d) Steps (b) to (c) are repeated, in order to improve the calculated parameters, by reducing the RMSE.

If, after several iterations (2 or 3 typically), the calculated RMSE is very high, the whole procedure must be repeated for different initial search intervals. This entire process ensures that the final fit parameters do not significantly depend on the first guess values.

## 3. Results and Discussion

### 3.1. Optical Parameters

In this section, the changes in *n* and *k* with increasing G loading for six P3HT/G nanocomposites are plotted, and finally, a fit for *n* and *k* is presented.

#### 3.1.1. Refractive Index and Extinction Coefficient

In the following section, the evolution of the complex refractive index of P3HT/G nanocomposites as a function of the wavelength and the influence of G content are discussed. For the calculations, experimental data for n and k for six nanocomposites with G concentrations in the range of 0–5 wt% have been taken [9].

**Figure 2:**
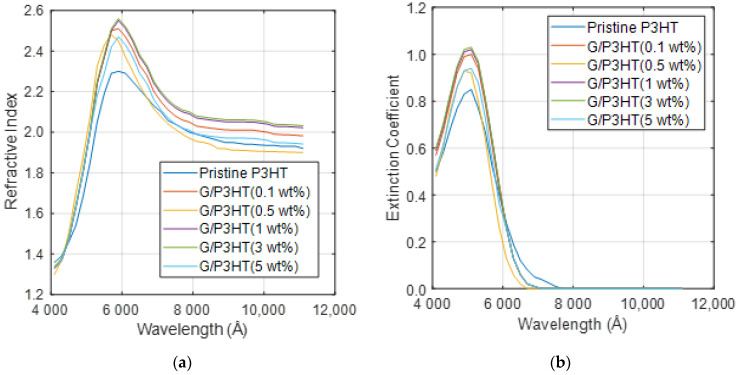
Refractive index (**a**) and extinction coefficient (**b**) obtained from ellipsometer analysis for P3HT/G nanocomposites with different G loadings. Adapted from Ref. [9], with permission from the American Institute of Physics, 2012.

Figure 2a shows the refractive index of these six nanocomposites. As can be observed, n increases with increasing G loading, except for the nanocomposite with 0.5 wt%, which shows a lower n value. Assuming a simple rule of mixtures [28], n should increase gradually with increasing G concentration, while experimental data reveal that the increment is more pronounced at low loadings and then tends to level off. At the peak wavelengths, it is observed that the optical constants increase with low graphene content and decrease slightly at higher G concentrations.

The change in the optical constants at low G contents suggests that this nanomaterial affects the optical properties of P3HT when it is well-dispersed throughout the polymer matrix. Likely, P3HT adheres to the G sheets, and due to the large surface area of this nanomaterial, the maximum change is observed at low loading, at which G agglomeration does not occur; hence, the maximum interaction takes place between P3HT and G [9]. The interaction likely occurs via π–π stacking between the π orbitals of the thiophene ring and the π orbitals of G, and it has been proposed that this interaction affects the planarity of P3HT. The closeness between the thiophen rings and G can lead to an excitation energy transfer from the P3HT donor to the G acceptor, as reported for P3HT and carbon nanotubes [29].

On the other hand, a maximum in *n* is detected at about 6330 A, which is consistent with the reported changes in the refractive index of G [30]. Thus, n of G varies from ~2.4 at 5320 Å to 3.0 at 6330 Å at room temperature. Similar behavior is found for *k*, as depicted in Figure 2b. The absorption coefficient of P3HT/G nanocomposites increases with increasing G loading, except for that with 0.5 wt% concentration, which shows lower k value than pristine P3HT. *k* is a measurement of how strongly a molecular species absorbs light at a given wavelength. However, it is common knowledge that the transmittance of a G sample depends on the number of stacked monolayers and is almost independent of the wavelength.

Overall, an increasing trend is found in both *n* and *k* with increasing G loading, which is in agreement with results reported previously for other polymer/graphene nanocomposites [31].

#### 3.1.2. Rule of Mixtures Applied to P3HT/G

The static optical properties of nanocomposite materials may be expressed in terms of the properties of their components using an appropriate, effective medium theory. The total differential model outlined here assumes that the materials do not undergo any phase changes and neglects stress effects. This is applicable to inorganic inclusions in a polymer host operating below their glass transition temperature. The rule of mixtures is presented as [28]:

(1) $$n_{m}=f\cdot n_{i}+\left(1-wt\right)\cdot n_{h}$$

where *n_m_*, *n_i_*, and *n_h_* are the real parts of the refractive indexes of the composite, inclusion, and host, respectively, and *wt* is the mass fraction of the inclusion. The original law considers the volume ratio instead of mass fraction. However, in the present study, the mass fraction instead of the volume ratio has been considered in order to avoid difficulties and errors derived from the conversion from mass fraction to volume fraction in a two-dimensional material such as graphene with an unknown volume density. Here, the host material is P3HT and the inclusion is G. n values of P3HT have been taken from the study of Saini et al. [9], and those of G have been adopted from Li et al. [32], who measured a trilayer graphene prepared via chemical vapor deposition.

It has been reported [28] that the rule of mixture describes the refractive index of polymer composites at low nanofiller loadings reasonably well, while noticeable differences can be observed at higher concentrations due to the effect of the coefficient of thermal expansion of the two materials on the volume fraction. Furthermore, the nanofiller particle size is a crucial factor. Accordingly, in order to fulfill the model, the nanofiller size should be small (i.e., less than 60 nm). This condition is fulfilled in the few layer-graphene used in the abovementioned studies [9,32].

Thus, at low loadings G is well dispersed throughout the polymer matrix, and the polymer can interact strongly with the G sheets, specially via π–π interactions [9]. However, at higher loadings, significant aggregation typically occurs, which prevents good G-polymer adhesion, and results in large nanofiller sizes, frequently in the order of microns; hence, strong deviations from theoretical predictions can occur.

**Figure 3:**
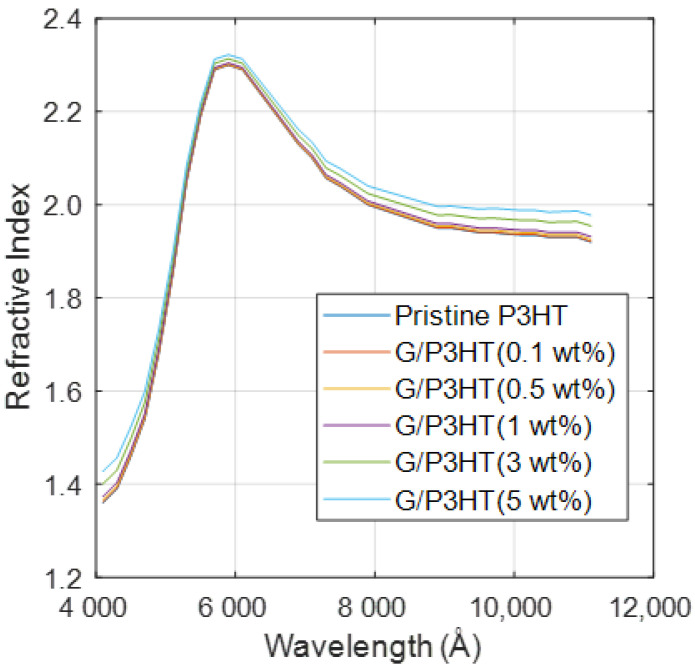
Refractive index of the six different P3HT/G nanocomposites calculated with the rule of mixtures.

The refractive indexes of the nanocomposites calculated with the rule of mixtures (1) are shown in Figure 3. The root mean square errors (RMSEs) between the values measured by Saini et al. [9] and those obtained with the rule of mixtures are presented in Table 2. The errors are quite small, lower than 6% in all the cases, even for the nanocomposite with 5 wt% G. According to Li et al. [31] the rule is not fulfilled for volume fractions of the inclusion higher than 10%.

Taking into account the results obtained, it can be concluded that the rule of mixtures can be successfully applied for n using mass fraction instead of volume fraction, thus avoiding the limitation of the two-dimensional nature of G and the difficulties in calculating its density in units of mass per volume.

The rule of mixtures was also applied to calculate the extinction coefficient of the nanocomposites, using the same models described for n, although the results obtained were not satisfactory. Thus, RMSEs between 30 and 42% were obtained for the calculated extinction coefficients of the five nanocomposites. These high values compared to those obtained for n are consistent with the literature [33]. In fact, most authors do not apply rules of mixtures for the estimation of the optical constants in absorbing mixtures because of the low accuracy obtained.

### 3.2. Forouhi Bloomer Fit for n and k

To fit the experimental data of the refractive index and the extinction coefficient of P3HT/G, a theoretical model that describes the behavior of *n* and *k* as a function of the wavelength or energy is needed.

The Forouhi Bloomer formulation is based on the quantum-mechanical theory of absorption. Crystalline semiconductors and metals may have several peaks in the shape of the curve of the extinction coefficient as a function of wavelength that reveal the presence of several oscillators. For an amorphous or crystalline material with a single oscillator [27], the expressions for *n* and *k* are:

(2) $$k\left(E\right)=\{\begin{array}{c} \frac{A\cdot {\left(E-E_{g}\right)}^{2}}{E^{2}-B\cdot E+C};forE>Eg\\ 0;forE\leq Eg \end{array}$$

(3) $$n\left(E\right)=\sqrt{\varepsilon_{\infty }}+\frac{B_{0}\cdot E+C_{0}}{E^{2}-B\cdot E+C}$$

where

(4) $$B_{0}=\frac{A}{Q}\cdot \left(\frac{-B^{2}}{2}+B\cdot E_{g}-E_{g}^{2}+C\right)$$

(5) $$C_{0}=\frac{A}{Q}\cdot \left(\left(E_{g}^{2}+C\right)\cdot \frac{B}{2}-2\cdot E_{g}\cdot C\right)$$

(6) $$Q=\frac{1}{2}\cdot \sqrt{4\cdot C-B^{2}}$$

As it can be observed in the equations, the parameters of n (*B*_0_ and *C*_0_) are dependent on the parameters of k, *A*, *B* and *C*. Therefore, there is no need to optimize *B*_0_ and *C*_0_, only *A*, *B*, *C* and $\varepsilon_{\infty }$ have been optimized in the fits shown below. The energy bandgap *E_g_* has also been optimized.

In Equations (2)–(6), A gives the amplitude of the extinction coefficient peak, *B*/2 is approximately the energy at which the extinction coefficient is maximized, and *C* is related to the broadening term of the absorption peak. $\varepsilon_{\infty }$ is the high-frequency dielectric constant. It should be higher than one and corresponds to the value of the dielectric function when ω→∞.

**Figure 4:**
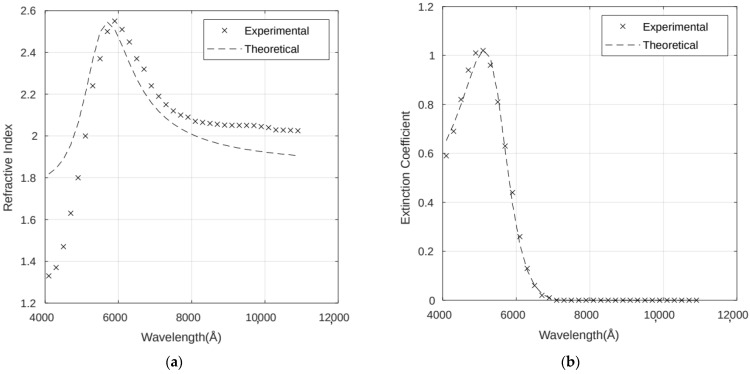
Refractive index (**a**) and extinction coefficient (**b**) fits to the Forouhi Bloomer model for the P3HT/G (1 wt%) nanocomposite. Experimental data are adapted from Ref. [9] with permission from the American Institute of Physics, 2012.

The complex refractive index for six P3HT/G nanocomposites with different G loadings has been fitted using the Forouhi Bloomer model. As an example, Figure 4a,b shows the fit for the P3HT/G (1 wt%) nanocomposite. The optimum parameters calculated for the six nanocomposites and the corresponding RMSEs are shown in Table 3. n and k are dependent on each other and require only five fitting parameters.

As it can be observed in Table 3, the errors lie between 7% and 9% for n and between 8% and 11% for *k*. In the case of n, the higher discrepancies between the calculated and the experimental values are in the wavelength range below 5000 Å. On the other hand, the discrepancies for *k* are more constant throughout the whole spectral range studied.

For all the nanocomposites, the bandgap is a fit parameter, and a value of 1.7 eV has been obtained compared to the theoretical one of about 2.0 eV. This discrepancy of 15% can be avoided by fixing the theoretical value in the fits at the expense of lower accuracy.

The discrepancies between the experimental and calculated values could arise from the fact that the refractive index of G is dependent on a number of factors such as flake thickness, size distribution, number of flakes, synthesis method and so forth [34,35].

In summary, the optical constants of the nanocomposites can be well described by the Forouhi Bloomer model based on the quantum-mechanical theory of absorption with only five fitting parameters. RMSEs lower than 11% are obtained in all the cases. As shown below, a higher accuracy is obtained with the Cauchy function at the expense of a higher number of parameters to fit.

### 3.3. Cauchy Fit for n and k

The earliest dispersion formula was established by Cauchy in 1836 [36]. The following equations connect the refractive index to the wavelength *λ* (in nm). Both *A_n_* and *A_k_* are dimensionless parameters. When *λ* tends to infinite, n tends to *A_n_*. *B_n_* and *B_k_*, in nm^2^, affect the curvature and the amplitude of the refractive index for medium wavelengths in the visible range. *C_n_* and *C_k_*, in nm^4^, affect the curvature and amplitude for smaller wavelengths in the UV range.

(7) $$n=A_{n}+\frac{10^{7}B_{n}}{\lambda^{2}}+\frac{10^{12}C_{n}}{\lambda^{4}}+\frac{10^{20}D_{n}}{\lambda^{6}}$$

(8) $$k=A_{k}+\frac{10^{7}B_{k}}{\lambda^{2}}+\frac{10^{12}C_{k}}{\lambda^{4}}+\frac{10^{20}D_{k}}{\lambda^{6}}$$

Cauchy’s formulation cannot be easily applied to metals and semiconductors. The parameters used do not have any physical meaning, and therefore, these empirical relations are not Kramers–Kronig consistent. From first principles, the Kramers–Kronig equation [37] relates the refraction index and extinction coefficient parts; it means that they are not independent quantities.

**Figure 5:**
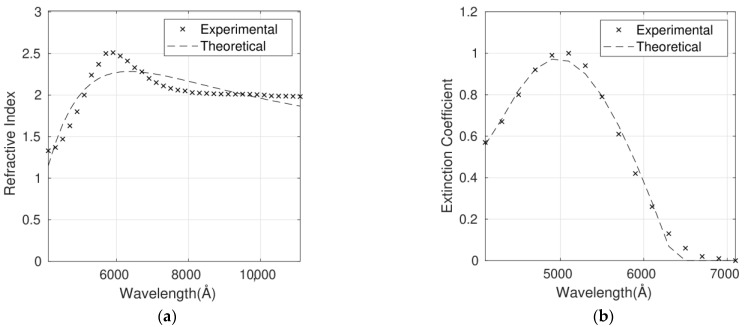
Refractive index (**a**) and extinction coefficient (**b**) fits to Cauchy’s equation for the P3HT/G (0.1 wt%) nanocomposite. Experimental data are adapted from Ref. [9] with permission from the American Institute of Physics, 2012.

**Figure 6:**
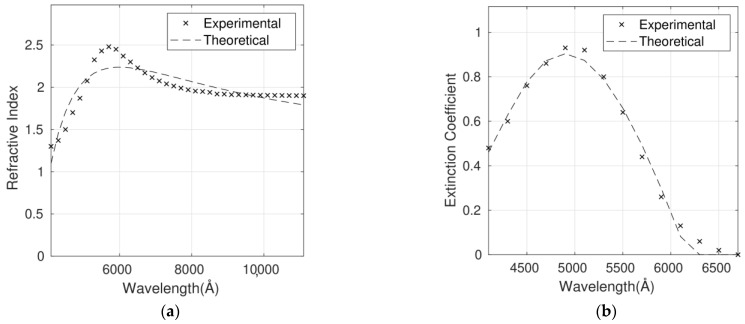
Refractive index (**a**) and extinction coefficient (**b**) fits to Cauchy’s equation for the P3HT/G (0.5 wt%) nanocomposite. Experimental data are adapted from Ref. [9] with permission from the American Institute of Physics, 2012.

**Figure 7:**
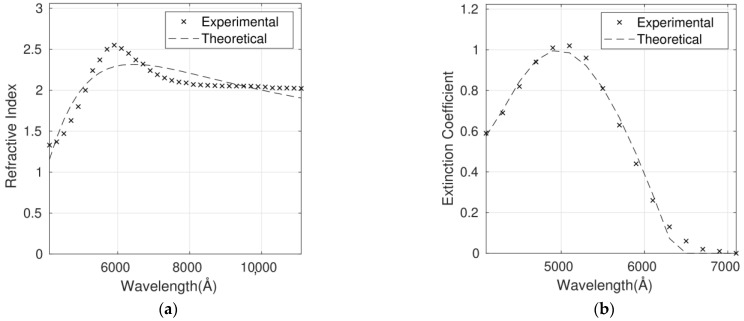
Refractive index (**a**) and extinction coefficient (**b**) fits to Cauchy’s equation for the P3HT/G (1 wt%) nanocomposite. Experimental data are adapted from Ref. [9] with permission from the American Institute of Physics, 2012.

**Figure 8:**
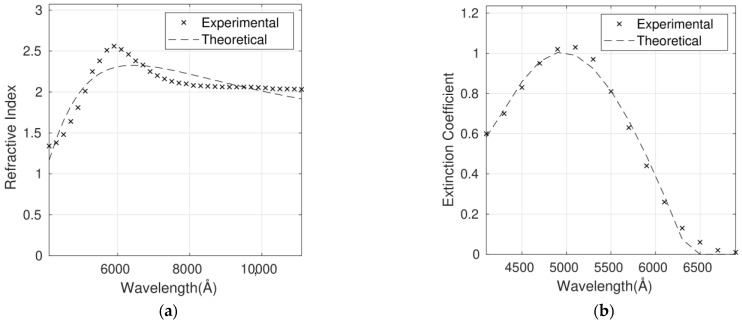
Refractive index (**a**) and extinction coefficient (**b**) fits to Cauchy’s equation for the P3HT/G (3 wt%) nanocomposite. Experimental data are adapted from Ref. [9] with permission from the American Institute of Physics, 2012.

**Figure 9:**
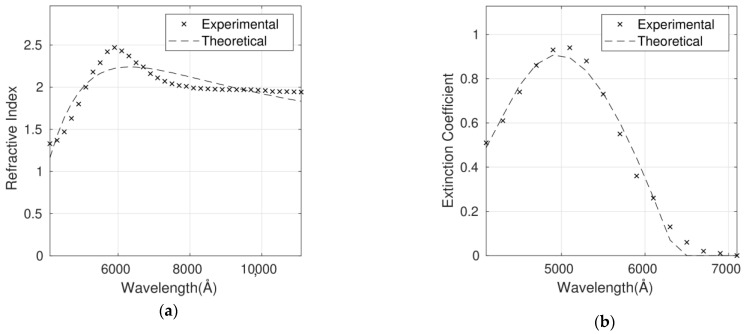
Refractive index (**a**) and extinction coefficient (**b**) fits to Cauchy’s equation for the P3HT/G (5 wt%) nanocomposite. Experimental data are adapted from Ref. [9] with permission from the American Institute of Physics, 2012.

**Figure 10:**
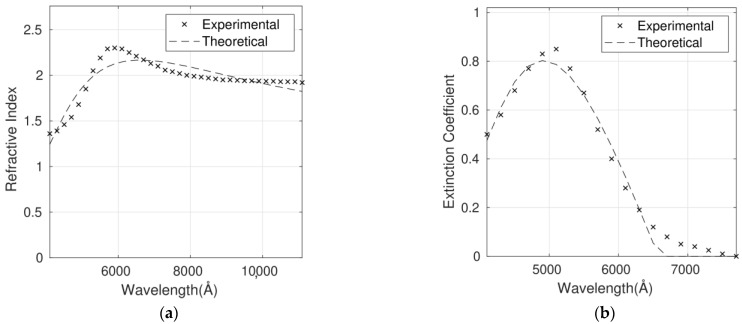
Refractive index (**a**) and extinction coefficient (**b**) fits to Cauchy’s equation for pristine P3HT. Experimental data are adapted from Ref. [9] with permission from the American Institute of Physics, 2012.

The complex refractive index for the six P3HT/G nanocomposites with different G loadings has been fitted using the Cauchy model (Figure 5, Figure 6, Figure 7, Figure 8, Figure 9 and Figure 10), and the optimum parameters calculated along with the RMSEs are shown in Table 4. *n* and *k* are independent and require four fitting parameters each, making a total of eight parameters for the fit. It is important to note that the errors are quite small, lower than 6% in the case of n and between 6% and 10% in the case of *k*. Both parameters are adequately described by this model in the whole wavelength range.

Although the Forouhi Bloomer model has more physical meaning and great potential, the Cauchy model leads to smaller errors and more accurate fittings (Table 3 and Table 4). Therefore, the RMSEs for *n* are about 50% higher for Forouhi Bloomer fits compared to Cauchy fits. In the case of *k*, RMSEs are also significantly higher for the Forouhi Bloomer model.

It is crucial to fit the experimental data to a model with the aim of creating a strong and simplified database. Overall, the comparison of the results obtained from the two models applied (Table 3 and Table 4) indicates that the Cauchy model is the most suitable for describing the optical properties of this type of polymer/graphene nanocomposite, which is a key factor in progress in their application in photovoltaic and optoelectronic devices.

## 4. Conclusions

In this work, a review of the changes in electrical conductivity, bandgap, hole collection properties and carrier mobility of P3HT (a conjugated polymer widely used in organic solar cells) upon addition of graphene has been presented. The conductivity, the hole collection properties and the carrier mobility are enhanced, while the bandgap is reduced with increasing G content. Experimentally, it has been found that both the refractive index (*n*) and the extinction coefficient (*k*) of P3HT increase with increasing G loading.

The optical constants n and κ of six P3HT nanocomposites with different G loadings have been fitted to two different models, Forouhi Bloomer and Cauchy, showing very good agreement between the experimental and the theoretical values.

Regarding the Forouhi Bloomer model, it has been shown that only five fitting parameters are required. Relative errors between 7% and 9% for n and between 8% and 11% for k have been obtained. On the other hand, the Cauchy model requires eight fitting parameters (four for *n* and four for *k*). Nevertheless, it leads to lower relative errors, smaller than 6% for n and in the range of 6–10% for *k*.

n of the nanocomposites has also been expressed in terms of the refractive index of each component using a rule of mixtures. Good results have been obtained incorporating the values of mass fraction instead of volume fraction, with RMSEs lower than 6% for all the nanocomposites studied.

Overall, the Cauchy model seems to be the most suitable for describing the optical properties of this type of polymer/graphene nanocomposites, which is a key factor in the progress of their application in organic solar cells and optoelectronic devices.

## Figures and Tables

**Table 1:** Conductivity measurements reported by Chang et al. for different P3HT/G nanocomposites. Adapted from [8], American Scientific Publishers, 2010.

Sample	Conductivity (S/m)
P3HT	9.273 × 10^−3^
P3HT/G 0.2 wt%	1.467 × 10^−2^
P3HT/G 1 wt%	1.549 × 10^−2^
P3HT/G 2 wt%	1.500 × 10^−2^
P3HT/G 10 wt%	1.878 × 10^−2^

**Table 2:** Root mean square error (RMSE) in the calculation of the refractive index of P3HT/G nanocomposites with different G loading.

G Loading (wt%)	RMSE (%)
0.1	4.87
0.5	4.86
1	5.72
3	5.49
5	3.04

**Table 3:** Optimal values of the fitting parameters to the Forouhi Bloomer model for six different P3HT/G composites. The root mean square errors (RMSE) of the fits are also shown.

G Loading (wt%)	Tabulated *Eg* (eV)	Optimized *Eg* (eV)	*A* (eV)	*B* (eV)	*C* (eV)^2^	$\varepsilon_{\infty }$ (eV)	n RMSE (%)	k RMSE (%)
0.1 wt%	2.0335	1.7	0.2730	4.4360	5.0200	3.1000	8.06	8.88
0.5 wt%	2.0659	1.7	0.1950	4.5980	5.3800	3.2800	9.08	10.08
1 wt%	2.0578	1.7	0.2790	4.4360	5.0200	3.2060	8.66	8.60
3 wt%	2.0555	1.7	0.2820	4.4360	5.0200	3.2200	8.61	8.40
5 wt%	2.0335	1.7	0.2490	4.4540	5.0600	3.0520	7.64	10.60

**Table 4:** Optimal values of the fitting parameters to Cauchy’s equation for six different P3HT/G composites. The root mean square errors of the fits are also shown.

G Loading (wt%)	Index	A	B (nm^2^)	C (nm^4^)	D (nm^6^)	RMSE (%)
0	*n*	1.2721	8.7069	−2490.000	170.7312	4.63
	*k*	−4.8152	33.6520	−6426.763	380.7333	10.40
0.1	*n*	1.3079	8.5623	−2138.199	110.0000	5.94
	*k*	−8.5932	60.0863	−12,177.988	783.9081	6.41
0.5	*n*	1.3460	6.4353	−1168.750	2.8906	5.87
	*k*	−8.9982	60.1133	−11,743.359	724.7188	6.72
1	*n*	1.3203	9.0273	−2349.000	132.0000	5.84
	*k*	−8.7457	61.1368	−12,379.167	796.1458	5.98
3	*n*	1.3305	9.0271	−2349.028	132.0000	5.81
	*k*	−8.5944	59.8669	−12,040.960	768.3500	5.98
5	*n*	1.2924	8.2404	−2039.611	103.8333	5.64
	*k*	−7.6083	52.7656	−10,511.359	660.5156	8.06

## Data Availability

The data presented in this study are available on request from the corresponding author.
